# A Stepwise Endovascular Approach to the Treatment of Refractory Plantar Fasciitis

**DOI:** 10.3390/healthcare14111562

**Published:** 2026-06-03

**Authors:** Piercarmine Porcaro, Ernesto Punzi, Andrea Izzo, Emanuele Flora, Enrico Maria Amodeo, Nobuaki Sakai, Giulio Lombardi

**Affiliations:** 1 Department of Radiology, “St. Giuseppe Moscati” Hospital of National Relevance and High Specialty, Contrada Amoretta, 83100 Avellino, Italy; piercarmine.porcaro@aornmoscati.it (P.P.); andrea.izzo@aornmoscati.it (A.I.); emanuele.flora@aornmoscati.it (E.F.); enrico.amodeo@aornmoscati.it (E.M.A.); giulio.lombardi@aornmoscati.it (G.L.); 2 Fukuoka Pain Care Clinic, 5-2-3 Nakasu, Hakata-ku, Fukuoka 810-0801, Japan

**Keywords:** plantar fasciitis, transcatheter arterial embolization, musculoskeletal embolization, chronic heel pain, TAME

## Abstract

**Highlights:**

**What are the main findings?**

- A stepwise, response-guided endovascular strategy achieved 100% technical success and was associated with substantial pain reduction in patients with refractory plantar fasciitis.
- Nearly half of patients improved after first-line minimally invasive embolization, while second-line TAME provided additional benefit in all non-responders.

**What are the implications of the main findings?**

- A staged approach allows tailored treatment intensity based on early clinical response, avoiding unnecessary invasive procedures in a substantial subset of patients.
- This strategy may represent a safe and cost-effective intermediate option between conservative therapy and surgery for chronic plantar fasciitis.

**Abstract:**

**Objectives**: To assess the feasibility, safety, and clinical effectiveness of a response-guided, stepwise endovascular treatment strategy for patients with refractory plantar fasciitis. **Methods**: This single-center retrospective study included consecutive patients with chronic plantar fasciitis refractory to conservative therapy who were treated between January and June 2025. All patients initially underwent ultrasound-guided direct puncture of the posterior tibial artery, followed by intra-arterial administration of imipenem/cilastatin as a temporary embolic agent. Clinical response was evaluated at 1 month using the visual analogue scale (VAS). Patients showing a <50% pain reduction were classified as non-responders and underwent second-line transcatheter arterial embolization (TAME) via transfemoral access, with selective embolization of pathological neovessels using bioresorbable microspheres. Technical success, pain outcomes, and procedure-related adverse events were assessed during follow-up for up to 6 months. **Results**: Twelve patients (13 treated feet) were included. First-line embolization was technically successful in all cases. At the 1-month follow-up, 6/13 feet (46.2%) demonstrated clinically meaningful pain reduction and required no further intervention. The remaining 7/13 feet (53.8%) underwent second-line TAME, which was technically successful in all cases and was associated with further pain reduction. Mean VAS scores decreased from 7.36 ± 1.12 at baseline to 1.37 ± 0.52 at 6 months. No major adverse events occurred; minor complications were self-limited. **Conclusions**: A stepwise endovascular treatment strategy for refractory plantar fasciitis appears feasible and safe, providing a high rate of symptom improvement while allowing procedural complexity to be escalated according to early clinical response.

## 1. Introduction

Plantar fasciitis is the most common cause of heel pain in adults and represents a significant clinical and functional problem within both the general and athletic populations [1,2]. It typically presents as localized calcaneal pain, often most intense during the first steps in the morning or after periods of inactivity. Several mechanical factors—including limited ankle dorsiflexion, excess body weight, abnormalities in foot arch structure, and repetitive high-impact activities—may contribute to its development [3]. Ultrasound has become a key diagnostic tool, allowing assessment of fascial thickening and increased vascularity, which are features commonly associated with symptom severity and functional impairment [4].

Although most patients respond to conservative management—including stretching, orthoses, physical therapy, shockwave therapy, corticosteroid injections, platelet-rich plasma, or prolotherapy [5,6,7,8]—a substantial proportion progress to a chronic, refractory form of the condition. In such cases, surgical treatment may be considered, but it is associated with variable outcomes, potential complications, and prolonged recovery times [9].

Recent advances have highlighted the role of pathological neovascularization in sustaining pain in chronic plantar fasciitis, prompting investigation of transcatheter arterial micro-embolization (TAME) as a targeted therapeutic option [10]. Abnormal neovessels may contribute to persistent symptoms by maintaining a local hypervascular environment associated with nociceptive signaling and, by analogy with other chronic musculoskeletal pain conditions, possible accompanying sensory nerve ingrowth. TAME has shown promising results in the treatment of osteoarthritic knee pain, suggesting a potential role for this minimally invasive technique in modulating nociceptive pathways associated with abnormal vascular proliferation. Initial reports in patients with chronic plantar fasciitis refractory to standard therapies have demonstrated high technical success rates and meaningful reductions in pain, without major ischemic complications [11,12,13].

These preliminary findings support the hypothesis that TAME may serve as an intermediate therapeutic option between conservative care and surgery. Most published studies [14,15,16,17] describe TAME for plantar fasciitis as a single-step procedure performed via transfemoral arterial access. In contrast, only limited reports have explored alternative strategies, such as multiple percutaneous posterior tibial arterial punctures [18], in some cases requiring several access sites per patient.

To balance efficacy with procedural invasiveness, we adopted a stepwise treatment strategy combining a simplified intra-arterial technique with conventional transcatheter embolization. The initial treatment consists of ultrasound-guided direct puncture of the posterior tibial artery, followed by intra-arterial administration of a temporary embolic agent (imipenem/cilastatin), with the aim of modulating pathological hypervascularity while minimizing access-related morbidity and technical burden. In patients without satisfactory clinical improvement at the 1-month follow-up, a second-line intervention is performed using a standard endovascular technique, including antegrade common femoral artery access, diagnostic angiography and selective embolization of vessels exhibiting pathological hypervascular blush.

The novelty of this approach lies in the integration of two previously described endovascular concepts into a structured, response-guided clinical pathway. Rather than applying conventional transcatheter arterial embolization as a single-step procedure in all patients, our strategy begins with a simplified ultrasound-guided posterior tibial artery infusion and reserves angiography-guided transcatheter embolization for patients with insufficient early clinical improvement. In this framework, procedural escalation is determined by clinical outcome at 1 month, allowing treatment intensity to be tailored to individual response while limiting more invasive interventions to non-responders.

## 2. Materials and Methods

### 2.1. Study Design and Patient Population

This single-center retrospective study included consecutive patients with chronic plantar fasciitis who underwent endovascular embolization at our institution between January 2025 and June 2025.

Plantar fasciitis was diagnosed based on clinical presentation and imaging findings, including persistent plantar heel pain localized at the medial calcaneal tubercle and ultrasound evidence of plantar fascia thickening (>4 mm) [4,19]. Inclusion criteria were persistent symptoms refractory to conservative treatment for at least 3 months, including physical therapy, orthotic support, non-steroidal anti-inflammatory drugs, or local injections.

Exclusion criteria included peripheral arterial disease of the affected limb, coagulation disorders, active local infection, prior foot surgery, rheumatoid or infectious arthritis, renal impairment (eGFR < 45 mL/min) or contraindications to endovascular procedures.

### 2.2. Ethical Considerations

According to institutional policies and in accordance with Article 110 of Italian Legislative Decree 196/2003 (Italian Privacy Code), as amended by Law No. 56/2024, retrospective observational studies based exclusively on anonymized data collected during routine clinical practice do not require formal ethics committee approval. Accordingly, this retrospective analysis was not submitted for separate ethics committee review. The embolization procedures were performed as part of standard clinical care. Written informed consent for the procedures was obtained from all patients, including specific discussion of the off-label use of imipenem–cilastatin. The off-label intra-arterial use of imipenem/cilastatin in this study was based on previously published clinical experience and reflects an established practice in musculoskeletal embolization aimed at achieving a temporary and controlled embolic effect. Given the retrospective nature of the study and the use of anonymized data, no additional consent for data analysis was required.

### 2.3. Treatment Protocol

**Figure 1:**
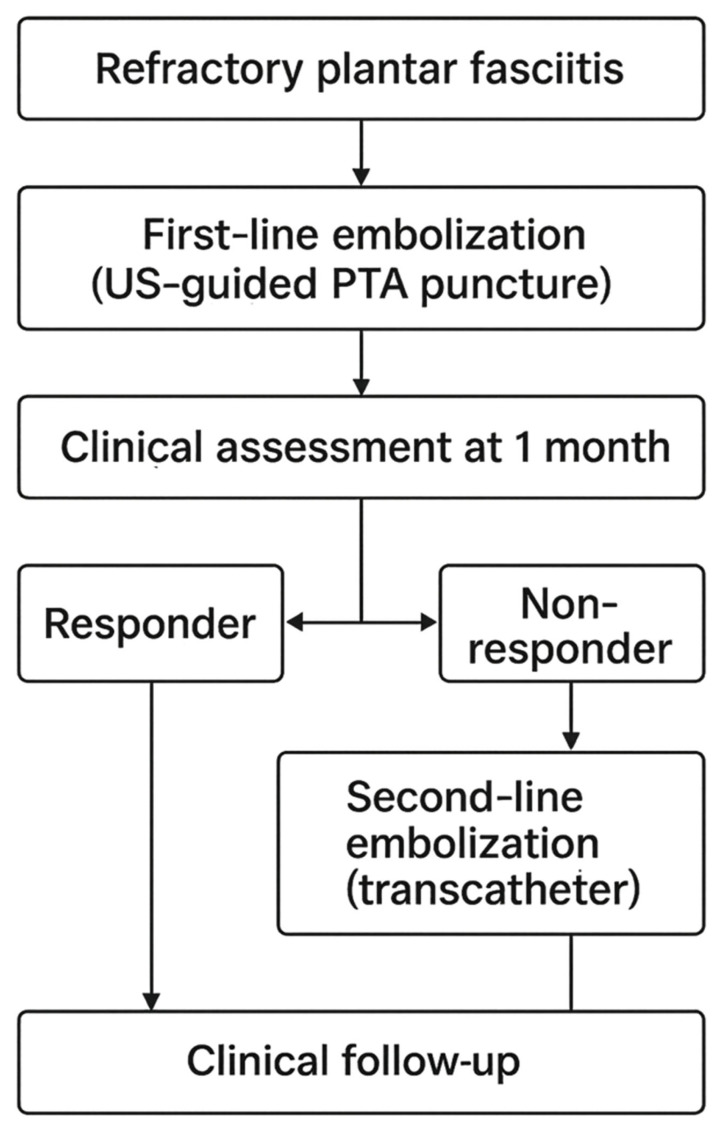
Stepwise endovascular treatment algorithm for refractory plantar fasciitis. The flowchart illustrates the predefined treatment strategy, including first-line ultrasound-guided posterior tibial artery embolization, clinical assessment at 1 month, and second-line transcatheter arterial embolization in non-responders.

All patients were treated according to a predefined stepwise clinical decision algorithm consisting of initial simplified intra-arterial embolization followed, when indicated, by conventional transcatheter arterial embolization (Figure 1).

All procedures were performed by four interventional radiologists with experience in ultrasound-guided vascular access and angiographic embolization.

#### 2.3.1. First-Line Procedure: Ultrasound-Guided Posterior Tibial Artery Embolization

Using an ultrasound scanner (Canon Xario 200G, Canon Medical Systems, Otawara, Japan) equipped with a 14L5 linear probe, the posterior tibial artery was punctured retrogradely with an in-plane approach using a 20-G needle (BD Spinal Needle, 20 G, Becton Dickinson, Franklin Lakes, NY, USA).

Under fluoroscopic guidance, 3 mL of non-ionic contrast agent (Iomeron 400 mg/mL, Bracco, Milan, Italy) was injected through the needle. The presence of a hypervascular blush in the calcaneal region—corresponding to the insertion of the plantar fascia—was confirmed.

**Figure 2:**
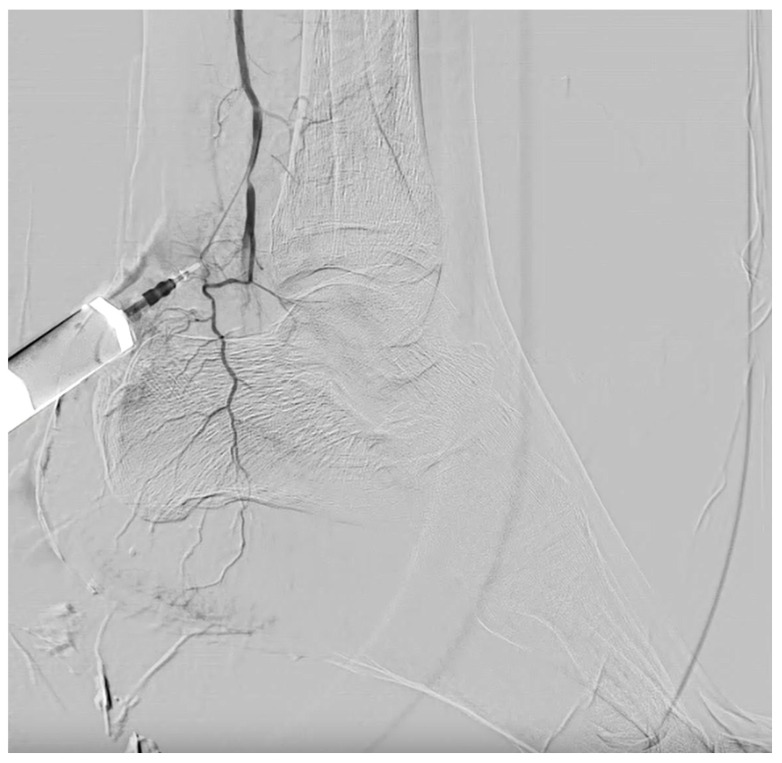
The fluoroscopic image obtained in the first-line procedure shows contrast injection through the needle positioned in the posterior tibial artery, confirming distal arterial opacification and hypervascular blush in the calcaneal region corresponding to the plantar fascia insertion.

Fluoroscopy was used only to confirm intraluminal needle position, distal contrast distribution, and the presence of a hypervascular blush, without selective catheterization or formal diagnostic angiography (Figure 2).

**Figure 3:**
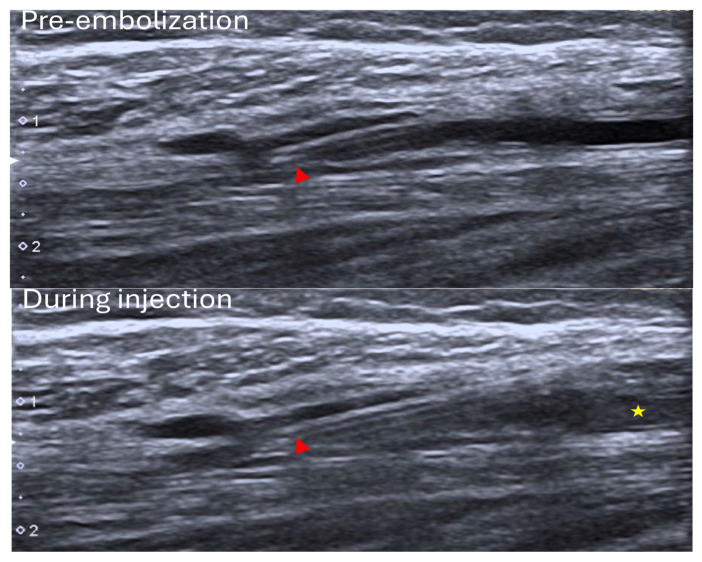
Ultrasound images acquired before embolization (**top**) and during imipenem–cilastatin injection (**bottom**). The needle tip is indicated by the red arrowhead. During injection, the appearance of intraluminal hypoechogenicity (yellow star) reflects the presence of imipenem/cilastatin, confirming intravascular delivery.

The temporary embolic agent IMP/CLS (imipenem/cilastatin) was prepared by diluting one vial in 10 mL of contrast medium. Because fluoroscopy was not used to monitor embolic material delivery or to define an angiographic embolization endpoint during first-line treatment, a standardized fixed volume of 2.0 mL of IMP/CLS suspension was administered uniformly in all treated feet through the needle positioned within the posterior tibial artery, followed by 1.0 mL of saline (Figure 3).

Transient skin blanching was observed at the calcaneal pain site, which resolved within approximately 10 min. At the end of the injection, the needle was removed, followed by 3 min of manual compression at the puncture site and an additional 10 min of clinical observation.

#### 2.3.2. Clinical Follow-Up and Criteria for Second-Line Treatment

Clinical evaluation was performed at the 1-month follow-up after the first-line procedure using visual analogue scale (VAS) [20].

Patients were classified as non-responders in the absence of satisfactory clinical improvement, defined as a <50% reduction in pain score compared with baseline [13]. Non-responders were scheduled for second-line angiographic treatment.

#### 2.3.3. Second-Line Procedure: Transcatheter Arterial Embolization

**Figure 4:**
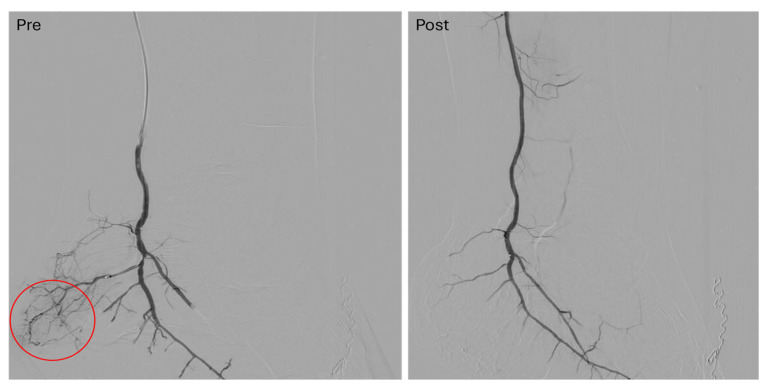
Digital subtraction angiography (DSA) images obtained before (**left**) and after (**right**) endovascular embolization. Baseline angiography demonstrates pathological vascular supply in the calcaneal region (red circle), while post-embolization angiography confirms occlusion of the pathological vessels with preservation of normal arterial flow.

Second-line treatment was performed using a standard transcatheter arterial embolization technique. Under local anesthesia, an ultrasound-guided antegrade puncture of the common femoral artery was performed. A 4-Fr vascular introducer (Cordis, Miami Lakes, FL, USA) was placed, and a 4-Fr BER II diagnostic catheter (Cordis) was advanced to the popliteal artery over a 0.035-inch hydrophilic guidewire (ZipWire, Boston Scientific, Marlborough, MA, USA). Using a 1.9-Fr Progreat Λ microcatheter (Terumo, Tokyo, Japan), the posterior tibial artery was catheterized distally to the calcaneal region. Diagnostic angiography of the distal lower-limb arteries was performed to identify target vessels, defined as arteries showing abnormal hypervascular blush at the calcaneal insertion of the plantar fascia (Figure 4) and/or reproducing the patient’s typical pain during selective contrast injection. Once pathological vessels were identified, selective embolization was performed using 100–300 µm bioresorbable microspheres (Nexsphere^®^, NextBioMedical, Incheon, Korea) diluted in 5 mL of normal saline and 5 mL of a high-osmolality iodinated contrast medium (Iomeron, 400 mg/mL, Bracco, Milan). Embolization was continued until complete disappearance of the hypervascular blush.

Arterial access was managed by manual compression (10 min), followed by 6 h of post-procedural monitoring. Ibuprofen 600 mg twice daily was prescribed for 3 days.

### 2.4. Outcome Measures, Safety Assessment and Statistical Analysis

Technical success was defined as successful delivery of the embolic agent to the target arterial territory. Clinical outcomes were assessed based on changes in visual analogue scale (VAS) score during follow-up.

The primary outcome measure was pain reduction assessed by VAS at the 6-month follow-up. Secondary outcomes included early pain response at 1 month, the need for second-line treatment, and procedure-related adverse events.

Procedure-related adverse events were recorded for both treatment steps and classified according to severity [21].

Given the exploratory nature of the study, the limited sample size, and the absence of a comparator group, analyses were primarily descriptive and no formal inferential efficacy testing was performed. Continuous variables were reported as mean ± standard deviation.

## 3. Results

### 3.1. Patient Population

A total of 12 patients (13 feet) were included in the study. The mean age was 50.4 ± 18.3 years, and 25% were female. The mean duration of symptoms prior to treatment was 17.4 ± 3.3 months. All patients had previously undergone conservative treatment without adequate clinical benefit. The mean pre-procedural VAS score was 7.36 ± 1.12.

Baseline demographic and clinical characteristics are reported in Table 1.

### 3.2. Feasibility and Technical Success of First-Line Treatment

Ultrasound-guided direct puncture of the posterior tibial artery was feasible in all patients. Technical success of first-line embolization was achieved in 13/13 treated feet (100%), with successful intra-arterial delivery of the embolic agent.

No immediate access-related complications or procedure interruptions were observed following the first-line treatment.

### 3.3. Clinical Response After First-Line Embolization

At the 1-month follow-up, the mean VAS pain score across all treated feet decreased from 7.36 ± 1.12 at baseline to 4.80 ± 2.25.

Overall, 5/12 patients (41.7%) and 6/13 treated feet (46.2%) achieved the prespecified clinically meaningful response threshold, defined as a ≥50% reduction in VAS pain score, and were classified as responders. In this group, mean VAS scores were 2.67 ± 0.52, 1.40 ± 0.54, and 1.40 ± 0.54 at 1-, 3-, and 6-month follow-up, respectively.

The remaining 7/12 patients (58.3%) and 7/13 feet (53.8%) did not achieve satisfactory symptom relief at the 1-month follow-up (mean VAS score 6.17 ± 1.72) and were classified as non-responders, according to predefined criteria. All non-responders at 1 month underwent second-line transcatheter arterial embolization.

### 3.4. Second-Line Treatment: Angiographic Findings and Embolization

Second-line transcatheter arterial embolization was performed in 7 non-responders. Diagnostic angiography demonstrated an abnormal hypervascular blush at the plantar fascia insertion and/or reproduction of concordant pain during selective contrast injection in all cases.

Target vessels most commonly originated from plantar arterial branches. Selective embolization was technically successful in all patients undergoing second-line treatment, with angiographic disappearance of the pathological blush at the end of the procedure.

### 3.5. Clinical Response After Second-Line Embolization

Following second-line treatment, further clinical improvement was observed in 7/7 (100%) patients. Clinical follow-up was performed at 1, 2, and 5 months after second-line embolization, corresponding to 2-, 3-, and 6-month follow-up after the initial first-line procedure. Mean pain scores progressively decreased from 6.17 ± 1.72 before second-line treatment to 3.00 ± 2.16, 1.67 ± 0.58, and 1.33 ± 0.58 at 2-, 3-, and 6-month overall follow-up time points, respectively.

The distribution of patients according to treatment step and clinical response is summarized in Table 2.

### 3.6. Overall Outcomes and Follow-Up

**Figure 5:**
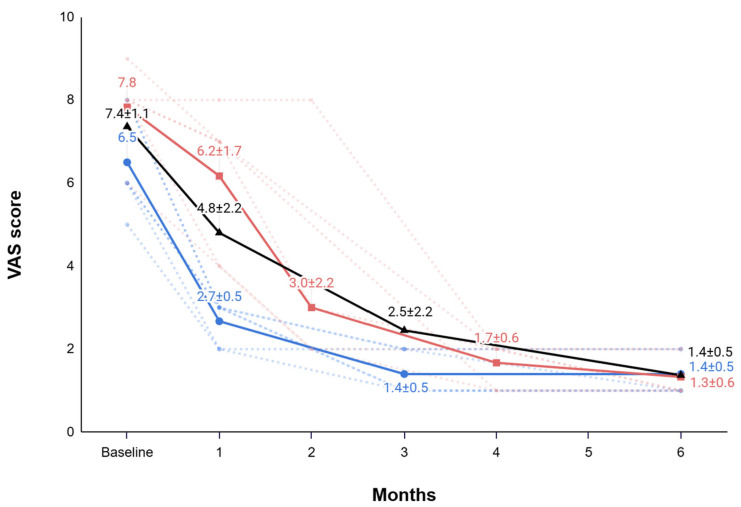
Individual patient data are shown as semi-transparent lines (blue for responders and red for non-responders). Thicker lines with solid symbols indicate mean values over time (blue circles for responders, red squares for non-responders, and black triangles for the entire cohort).

When the two-step escalation strategy was applied—initial percutaneous intra-arterial treatment with crossover to angiography-guided embolization for non-responders at 1 month—all patients (12/12) and all treated feet (13/13) showed clinically meaningful pain reduction at the final 6-month follow-up. Mean VAS scores decreased from 7.36 ± 1.12 at baseline to 4.80 ± 2.25, 2.45 ± 2.19, and 1.37 ± 0.52 at 1, 3, and 6 months, respectively. The temporal evolution of pain scores during follow-up is illustrated in Figure 5.

### 3.7. Safety and Adverse Events

No major adverse events occurred during either treatment step. Minor complications included a transient, self-resolving subcutaneous hematoma at the puncture site in one patient after second-line treatment and post-procedural calcaneal pain in one patient following the transcatheter approach; the latter required non-steroidal anti-inflammatory drugs for 2 days and resolved completely without further complications. No cases of vasospasm related to direct posterior tibial artery puncture were observed.

## 4. Discussion

The main finding of this study is that a stepwise endovascular approach to the treatment of refractory plantar fasciitis was associated with a high overall rate of clinical improvement at 6-month follow-up. A substantial proportion of patients experienced meaningful symptom relief after the initial simplified intra-arterial embolization alone, while additional benefit was observed in non-responders treated with second-line transcatheter arterial embolization.

From a clinical perspective, this approach functions as a decision-making pathway rather than a purely technical modification, using early clinical response to guide further intervention. Previous reports on arterial embolization for plantar fasciitis have predominantly described single-step transcatheter approaches [14,15], whereas the contribution of the present study is primarily algorithmic: it proposes a response-guided escalation strategy rather than a single uniform embolization technique for all patients. By offering a minimally invasive, ultrasound-guided intra-arterial treatment as a first step, this strategy may allow a substantial subset of patients to be managed without transfemoral access or catheter-based angiography, potentially reducing procedural complexity and resource use [18]. For non-responders, second-line transcatheter embolization enables angiography-guided, targeted treatment of pathological vessels. This escalation strategy may avoid repeated empirical direct-puncture treatments in patients with insufficient response to the initial simplified procedure. Although the present study was not designed to compare this stepwise strategy with single-step TAME, the observed outcomes suggest that favorable intermediate-term results may be achievable while reserving conventional transcatheter embolization for selected patients.

Nevertheless, direct posterior tibial artery puncture may present technical limitations, including needle displacement during injection and puncture-related vasospasm, although neither event occurred in our cohort.

In addition, the prolonged symptom duration in our cohort (mean, approximately 17 months) suggests that the study population has established chronic refractory symptoms at the time of the treatment, supporting the interpretation that the observed clinical benefit is not readily attributable to the natural history of the disease.

The differential response to first-line treatment may reflect heterogeneity in the extent and distribution of pathological neovascularization among patients. In cases where abnormal vascular supply is limited or accessible through posterior tibial artery branches, direct intra-arterial embolization may be sufficient to achieve symptom relief. Conversely, patients requiring second-line treatment may present with more complex or diffuse vascular patterns, which are more effectively addressed through selective transcatheter embolization under angiographic guidance. This escalation step allows direct identification of residual pathological hypervascularity and enables superselective embolization of target vessels, thereby providing a mechanistically more targeted option for non-responders.

This approach was associated with a favorable safety profile, with no major procedure-related adverse events observed. The use of a temporary embolic agent and the avoidance of transfemoral access in the first-line procedure may contribute to minimizing access-related and ischemic complications.

This study has several limitations. Its single-center design and relatively small sample size reduce statistical power, limit the precision of outcome estimates, and restrict the generalizability of the findings to broader patient populations. This limitation is particularly relevant in the context of a response-guided stepwise strategy, because stratification into responders and non-responders after first-line treatment results in very small subgroups and prevents robust subgroup-level comparisons or identification of predictors of response. In addition, because this was a retrospective study describing a treatment pathway adopted in routine clinical practice, no contemporaneous control or comparator group receiving conservative therapy or single-step TAME was available. Clinical outcomes were primarily assessed using patient-reported measures, which may be subject to reporting variability. Moreover, although a 50% reduction in pain was selected as a clinically stringent responder threshold, this cutoff has not been specifically validated for plantar fasciitis embolization and therefore remains partly operational; alternative thresholds may influence responder classification. Finally, the 6-month follow-up period allows assessment of short- to intermediate-term outcomes but is insufficient to establish long-term durability, recurrence rates, or the need for retreatment.

An additional consideration is that the two treatment steps differed not only in access route and imaging guidance, but also in the embolic material used. IMP/CLS was selected for the first-line procedure because of its very short-lasting embolic effect, which was considered more appropriate for a relatively non-selective posterior tibial artery infusion through direct puncture, potentially limiting the risk of non-target or prolonged distal ischemia. Conversely, second-line embolization was performed using 100–300 μm bioresorbable microspheres, which provide a more sustained, although still temporary, embolic effect. Their use was considered appropriate in the setting of angiography-guided superselective catheterization, which allows more targeted embolization of pathological vessels in non-responders. Therefore, differences in embolic duration may have contributed to the outcomes observed after second-line treatment. The present study was not designed to compare embolic agents, but to explore a response-guided treatment pathway in which procedural selectivity and embolic durability were escalated together.

Despite these limitations, the findings of this study support the feasibility of a stepwise endovascular treatment algorithm for refractory plantar fasciitis. Future prospective studies with larger cohorts, control groups, and longer follow-up are warranted to confirm these results, assess durability and recurrence, refine patient selection criteria, and better define the role of simplified intra-arterial embolization within the therapeutic algorithm of chronic plantar fasciitis.

## 5. Conclusions

This staged strategy may represent a practical endovascular treatment pathway for refractory plantar fasciitis, allowing escalation of procedural complexity only when clinically required.

In a substantial proportion of patients, meaningful clinical improvement was achieved with first-line ultrasound-guided posterior tibial artery embolization alone, while second-line transcatheter treatment provided additional benefit in non-responders, resulting in a high overall rate of symptom relief at 6-month follow-up.

Further prospective studies with larger cohorts and longer follow-up are warranted to confirm these findings and to better define patient selection criteria.

## Figures and Tables

**Table 1:** Baseline demographic and clinical characteristics of patients with refractory plantar fasciitis undergoing stepwise endovascular treatment. VAS: visual analogue scale; ESWT: extracorporeal shock wave therapy. Values are expressed as mean ± standard deviation or number (percentage), as appropriate.

Variable	Value
Number of patients	12
Number of treated feet (plantar fasciitis)	13
Age (years), mean ± SD	50.4 ± 18.3
Female sex, *n* (%)	4 (25%)
Symptom duration (months), mean ± SD	17.4 ± 3.3
Affected side (right/left), *n*	6/7
Previous conservative treatments, *n* (%)	13 (100%)
– Physical therapy	11 (84.6%)
– Orthotic support	9 (69.2%)
– Local injections (e.g., corticosteroids; platelet-rich plasma (PRP) injection)	7 (53.8%)
– Extracorporeal shock wave therapy (ESWT)	9 (69.2%)
Baseline VAS score, mean ± SD	7.36 ± 1.12

**Table 2:** Clinical outcomes and pain evolution during follow-up in patients with chronic plantar fasciitis treated with a stepwise embolization strategy. All feet underwent first-line embolization, with second-line treatment reserved for non-responders. VAS scores are reported as mean ± standard deviation.

Variable	Value
Numbers of plantar fasciitis treated with first-line embolization	13
Baseline VAS score, mean ± SD	7.36 ± 1.12
VAS score at 1-month follow-up after first-line treatment (all patients)	4.80 ± 2.25
Clinical responders after first-line treatment	6/13 (46.2%)
VAS score in responders at 1-, 3-, and 6-month follow-up	2.67 ± 0.52 1.40 ± 0.54 1.40 ± 0.54
Non-responders undergoing second-line embolization	7/13 (53.8%)
VAS score in non-responders before second-line treatment	6.17 ± 1.72
VAS score in non-responders at 1-, 2-, and 5-month follow-up after second-line treatment	3.00 ± 2.16 1.67 ± 0.58 1.33 ± 0.58
Overall clinical responders at 6-month follow-up	13/13 (100%)
Overall VAS score at 1-, 3-, and 6-month follow-up after the stepwise treatment strategy	4.80 ± 2.25 2.45 ± 2.19 1.37 ± 0.52

## Data Availability

The data presented in this study are available on request from the corresponding author due to patient privacy and institutional data protection restrictions.

## Abbreviations

The following abbreviations are used in this manuscript:

IMP/CLS	Imipenem/cilastatin
TAME	transcatheter arterial micro-embolization
VAS	visual analogue scale
